# Oviposition and Development of *Anopheles coluzzii* coetzee and Wilkerson in Salt Water

**DOI:** 10.1155/2019/9523962

**Published:** 2019-10-09

**Authors:** E. K. Nwaefuna, Ibalafake Ibisobia Bagshaw, F. Gbogbo, M. Osae

**Affiliations:** ^1^Radiation Entomology and Pest Management Centre, Ghana Atomic Energy Commission, Ghana; ^2^Department of Animal Biology and Conservation Science, University of Ghana, Ghana

## Abstract

*Anopheles coluzzii* is an important vector of malaria in sub-Saharan Africa particularly of the most dangerous malaria parasite. It completes its life cycle in water and a change in physicochemical properties particularly that of salinity of water may affect egg laying and perhaps the development of eggs to maturity. Studies have shown that climate change may alter the transmission of many vector-borne diseases in different parts of the world and global warming will also raise sea levels which will lead to an increase in saline and brackish water body in coastal areas. This study investigated the salinity tolerance level of *An. coluzzii.* It involved creation of artificial environments of different salinity gradients using rainwater and sea water and the subsequent exposure of the media to *An. coluzzii* for laying of eggs and development of larvae to adult. *Anopheles coluzzii *showed ovipositional preference for less saline media as there was significant negative correlation between number of eggs laid and salinity of oviposition media. Effect of salinity was evident in egg development and larval survival, as no egg hatched in >30% sea water, all L3 larvae died in >40% seawater, and the maximum seawater concentration for L4 survival was 30%. An L_C_50 of 17.51% (95% CI: 9.31–24.56)% and 23.4% (95% CI: 16.76–22.30)% were calculated for L3 and L4 larvae respectively. Adults emerging from fresh and low saline water of 10% seawater had greater energy reserve than those emerging from 20% and 30% seawater. Increasing salinity did not affect wing length of the emerging adult. Despite the increased stress on larval development, some individuals survived and went on to emerge as adults in conditions that seem to be representative of brackish water. This may imply that an increase in brackish water sites caused by rising sea levels might create more suitable breeding sites for this species.

## 1. Background

Malaria is the most devastating vector-borne disease worldwide and is transmitted by anophelines of which *Anopheles coluzzii *is one of the main vectors of malaria in Ghana. Salinity tolerance is the physiological and molecular mechanism of tolerance to osmotic and ionic components of salinity stress [1]. Salt water tolerance in mosquitoes is a trait that carries both ecological and epidemiological implications and plays a key role in determining habitat use and ecological distribution and thus local contribution of mosquitoes to disease transmission [2]. *Anopheles coluzzii* like every other organism has a minimum and maximum level of salinity stress which they can tolerate, but its larvae are generally considered to inhabit sunlit, shallow, temporary bodies of freshwater [3]. However, there is evidence this species is becoming adapted to habitats such as polluted water pools, which hitherto were not associated with, due to ecological changes [4, 5].

Climate change is resulting in ecological changes with consequent changes in the range of species. There is ample evidence that the habitat range of most mosquito species is changing as a result of climate change [6–8]. Climate seems to be the most important natural cause of salinization of inland freshwater bodies with consequence impact on the availability and distribution of freshwater [9]. Only about 2% of the world's water consist of freshwater with about 70% of the freshwater existing in the form of ice [10]. Climate change has an adverse effect on this proportion with the prominent effect being the continuous melting of glaciers in the Polar Regions into the sea [10]. Thus, as expected, the availability of freshwater may actually decrease because increased ocean volume will cause sea levels to rise, contaminating freshwater sources along coastal regions with seawater [9]. Thereby, increasing the availability of brackish water sites.

Global climate change can potentially increase the transmission of mosquito-borne diseases like malaria, lymphatic filariasis and dengue in people inhabiting coastal areas if these vectors become adapted to surviving in saline water [11]. Under laboratory conditions, *An. coluzzii* showed discrimination against sea water, though the larvae demonstrated some level of salinity tolerance [12]. In deed in nature, *Anopheles *species have been found breeding in water with salinity up to 30% (10.5 g NaCL/L) [13]. Based on different scenarios of climate change, it has been established that there could be increase in seawater intrusion particularly in the rivers and streams in the low-lying coastal areas of Ghana if the sea levels continue to rise [14]. It is thus important to evaluate the levels of tolerance of local populations of the major mosquito disease vectors to salinity and for that matter, the effect of climate change on mosquito-borne disease transmission in Ghana. This paper reports studies that sought to determine the oviposition and developmental preference of one of the major malaria vectors (*An. coluzzii*) in Ghana.

## 2. Methodology

### 2.1. Study Site and Mosquito Colony

All laboratory experiments were carried out at the insectary of the Radiation Entomology and Pest Management Centre, Ghana Atomic Energy Commission (GAEC) between November 2014 and March 2015. The *An. coluzzii* colony used for the experiments was established in November 2014 from larval collections from Okyereko (05° 24.960 N 000° 36.729”W), an irrigation rice producing village in the Central Region of Ghana.

Anopheles larvae and pupae were collected from the rice fields and brought to the insectary for rearing. Rearing and all experiments were carried out at 27 ± 2°C temperature, 75 ± 5% relative humidity, and a photoperiod of 12 : 12 (light: dark). Larvae were fed with powdered fish meal (Lopis Goldfish^TM^, Martin and Martin Pty. Limited, Kempton Park, South Africa) until pupation. Emerged adults were fed with 10% glucose solution in cotton balls and females provided with blood meal from the forearm of the researcher for 15 minutes. The individuals used for all experiments ranged from F_2_ to F_4_ generations of the collections.

### 2.2. Species Identification

Iso-female egg-laying cups were set up for individual female gravid mosquitoes. DNA was extracted from the parents and PCR run to identify the species according to [15]. Only offspring from parents confirmed to be *An. coluzzii* was added to the colony and used for the study.

### 2.3. Saline Water Preparation

Seawater considered to have 100% salinity (38.1 ppt of salt) was collected from the Nungua beach along the coast of Greater Accra Region of Ghana and serially diluted by addition of rainwater (0 ppt of salt) to range from freshwater (0% seawater) to saline water (100% seawater) with increment of 10%. The physiochemical parameters (salinity, temperature, dissolved oxygen, total dissolved solid, pH, conductivity) of the different dilutions were measured using Horriba U50 multi-parameter probe by submerging it into the various dilutions and measurements recorded. The water samples were stored in clean 4 liter containers in the refrigerator at 4°C until ready to be used. Prior to use, the water was removed and allowed to stand on the laboratory bench overnight.

### 2.4. Effect of Salinity on Oviposition, Hatchability and Larval Development

A total of 200 blood-fed female *An. coluzzi* were introduced into each of ten (10) 30 × 30 × 30 cm bugdom™ cages (MegaView Science, Taiwan). After 48 hours, oviposition cups containing water with the different salinities prepared were placed in the cages and allowed 24 hours for oviposition. The oviposition cups used were plastic, white in colour, 3 cm in length and 3 cm in diameter, and lined with filter paper. The eggs were held in the oviposition cups for 72 hours, under standard insectary conditions (27 ± 2°C temperature, 75% ± 5 humidity and a 12 h: 12 h light: dark cycles) after which hatchability was determined. The entire content of each oviposition cup was sieved through a whatman No. 1 filter paper; eggs and larvae were counted under a stereoscope to determine the proportion that hatched. The experiment was replicated four times and average eggs laid and percentage hatched calculated for each medium.

Salinity tolerance of larvae was evaluated using second, third, and fourth instars. For each larval stage, 30 larvae were introduced into water at each of the salinity levels in 500 ml ice cream cups and fed daily as described above till they pupated and developmental time recorded. The pupae were collected and allowed to emerge into adults in paper cups covered with nets and kept for further studies. The larval experiments were repeated four times for each larval stage, average pupation, and emergence calculated.

### 2.5. Determination of Energy Reserve and Morphometric Measurment

Adult *An. coluzzi* that emerged from the salinity tolerant test were used to determine energy reserve by leaving them in 350 ml paper cups covered with nets and starved to observe how long it took for them to die. The mosquitoes were maintained under standard insectary conditions as stated above. Survival time was used as a proxy for determination of energy reserves.

Dead adults were stored in containers with silica gel and preserved in a refrigerator for further studies. Wing length was measured using a Leica Application Suite (LAS) EZ microscope (version 2.1.0) with an inbuilt camera to determine effect of salinity on the morphology of the adult. Emerged adults from each salinity level were picked at random and wings dissected carefully using a dissecting pin and mounted on a microscope slide. Wing length was measured as a straight line from the base of the axillary area to the apex of the right wing at ×35 magnification. The LAS software was used to determine wing measurements.

## 3. Data Analysis

The data were tabulated appropriately and entered into Statistical Package for Social Scientists (SPSS) software (version 18). Data on egg production, hatchability, larval developmental time, pupation, energy reserves as well as morphometric measurements were analyzed by ANOVA and Tukey's HSD test for mean comparison where there were significant differences. Log-dose-probit analysis was used at a confidence level of 95% to calculate the LC-50 for both L3 and L4 larvae using SPSS version 18. Pearson's correlation analysis was used to determine relationship between salinity and oviposition preference. All analyses were done at the 0.05 confidence level.

## 4. Result and Discussion

### 4.1. Physicochemical Parameters

**Figure 1:**
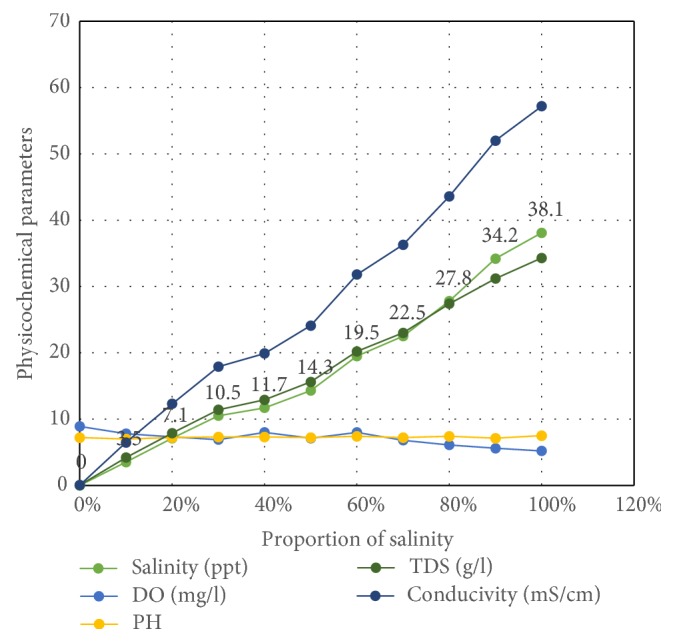
Variations in some physicochemical parameters in serial dilutions of seawater.

Though a wide range of variations occurred in the physicochemical properties of the various media prepared from varying proportions of fresh and saltwater samples, pH and dissolved oxygen (DO) did not change significantly with sea water dilutions from 0 to 100%. Both the pH and DO remained within the range recorded from natural breeding sites of *An. ganbiae* s.l., [16] but as expected, TDS and salinity increased from 0.025 g/l and 0.02 ppt to 34.3 g/l and 38.1 ppt in freshwater and 100% seawater, respectively. There was a direct linear relationship between TDS and salinity, as the dissolved ions are from the salts [17]. Levels of TDS and the combinations of ions impact aquatic life to varying degrees [18]. Thus, the increased TDS and salinity at higher concentrations of seawater was expected to negatively impact survival of *An. coluzzii* since this species is not known to be adapted to high levels of ion concentration (Figure 1).

### 4.2. Effect of Salinity on Oviposition and Egg Hatchability

**Figure 2:**
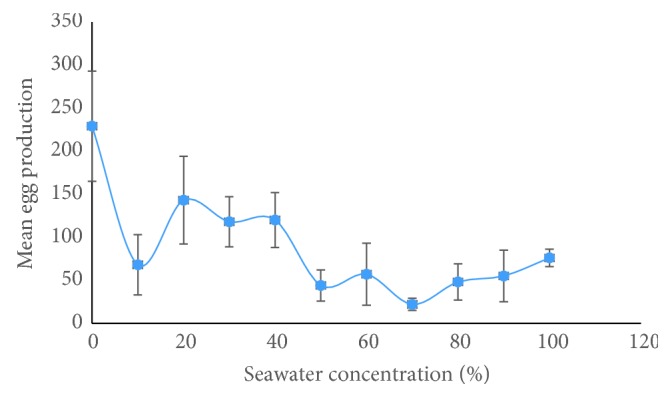
Oviposition preference of* An. Coluzzii* for water with different salinity levels expressed as percentage seawater concentration.

Female *An. coluzzii* laid eggs across a wide range of salinities than was expected. About 60% of total eggs were laid in water with salinity ranging from 0 ppt (freshwater) to 11.7 ppt (40% seawater). Pearson's Correlation analysis showed a significant negative correlation (*r* = 0.54, *p* = 0.001) between salinity and oviposition preference of *An. coluzzii* indicating that as salinity increases females are less likely to lay eggs in water. It is known that gravid female mosquitoes depend on environmental cues to select oviposition sites and the ability to detect a suitable site is a critical trait in most species [19]. Thus, the decrease in oviposition with increasing salinity reflects a deliberate avoidance of unsuitable oviposition sites by this species. However, the fact that some eggs were deposited in all the varying salinity levels including 100% seawater indicates that other factors might be at play in the selection of oviposition sites (Figure 2). The use of nochoice tests in this study is very relevant because brackish water sites are likely to cover extensive areas leaving mosquitoes without much of a choice of breeding sites.

Salinity also had a significant effect on egg hatchability. Eggs hatched in the control (freshwater) was 73%, decreasing significantly (*P* = 0.007, *F*_32_ = 3.516) to 0.4% in the 30% seawater dilution, beyond which there was no hatching. These findings are similar to previous reports indicating that *An. gambiae *showed oviposition preference for freshwater as compared to other saline water bodies [20, 13].

Despite the fact that in this study the mosquitoes laid eggs in all of the water samples their oviposition preference for fresh water was clear. This result slightly differs from the findings of [21] reporting that Anopheles mosquitoes discriminatingly lay eggs in preferred fresh water bodies. This difference may have been as a result of laboratory conditions.

**Figure 3:**
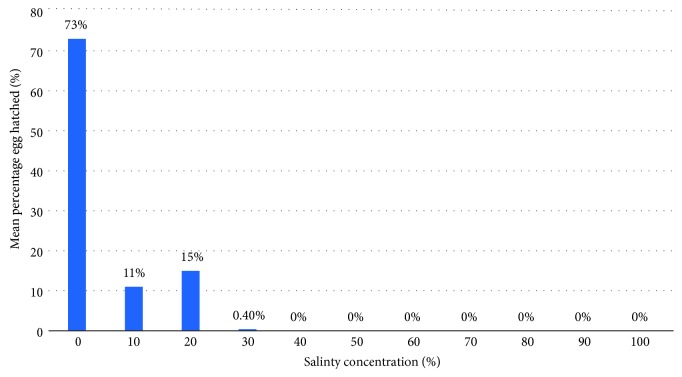
Mean percentage of egg* An. Coluzzii* eggs hatched in different saline concentrations.

Salinity affected egg development as egg hatchability declined with increasing seawater concentration up to 30% seawater (10.5 ppt) where no egg hatched. It has also been observed that salinity level of 15.85 g NaCl per liter equivalent to 50% salinity killed all *Anopheles gambiae *[22] which is similar to our observation that no *An. coluzzii* larvae survived above 30% seawater. This implies that even if eggs are deposited in saline water, they may not increase malaria risk. However, the fact that some eggs hatch in water containing up to 30% (10.5 ppt salinity) means this species has the potential to adapt to brackish water with consequent increase in the range of such an efficient malaria vector (Figure 3).

### 4.3. Effect of Salinity on Larval Development

**Figure 4:**
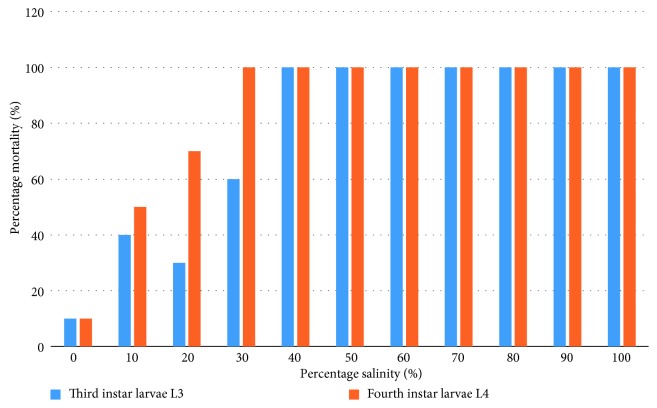
Dose mortality response of third and fourth instar larvae to salinity exposure.

Third and fourth instars (L3 and L4 respectively) showed salinity dependent mortality. No L3 larve survived in rearing media containing 40% seawater and above while the minimum seawater concentration for L4 survival was 30%. An L_C_50 of 17.51% (95% CI: 9.31–24.56)% sea water was calculated for the L3 larvae, while L_C_50 for the L4 larvae was 23.4% (95% CI: 16.76–22.30)%. There was significant difference between the mortalities recorded in the control and those recorded in all the media that had seawater (Figure 4).

**Figure 5:**
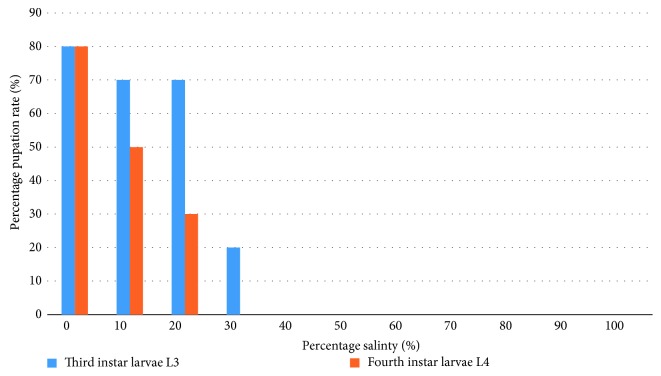
Percentage pupation rate of third and fourth instar larvae in salinity exposure.

For L3 larvae, 80% pupated in the control media while 70% pupated in 10% and 20% salinity with not more than 20% larvae pupating in 30% salinity (Figure 5). Pupation of L4 larvae occurred only up to 20% seawater with as low as 50% and 30% pupation in the 10% and 20% seawater media, respectively, compared to 80% pupation in the control medium. Salinity tolerance depends on the ability of the organism to osmoregulate by possessing organs for eliminating excess ions from the hemolymph and producing concentrated urine or osmoconform by accumulating high levels of osmolytes in the hemolymph in response to increased environmental salinity [23, 24]. Some salt-tolerant larvae of *Aedes spp* are known to osmoregulate and possess additional rectal segments that serve as sites of active transport [24], whereas the osmoconforming larvae of *Culex tarsalis* accumulate high levels of proline and trehalose in the hemolymph [22]. *An. coluzzii* may not possess any structures for osmoregulation, but the fact that some larvae are able to develop to pupation in up to 20% seawater is a concern and require further studies to determine whether they are capable of osmoconformation. This is most important for *An. coluzzii* which is known to breed in rice fields where soil salinization is a common phenomenon (Figure 5). Pupation rate was observed to be higher when larvae were introduced as L3 compared to when introduced at L4 to increasing saline concentrations; however, survival of the L3 was lower as compared to L4. This may be a pointer to the degree of toxicity of salt resulting from the longer exposure of the L3 as compared to the shorter exposure of the L4 to salinity.

### 4.4. Energy Reserve

**Figure 6:**
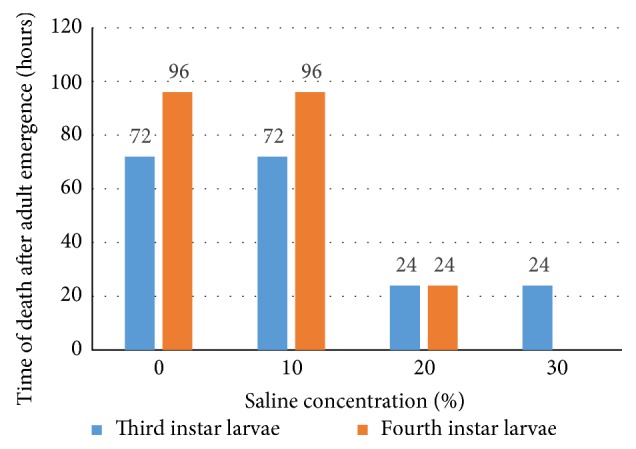
Survival time used as proxy for energy reserve in adult emerged from different saline concentration.

Mosquitoes emerged from control and mild saline concentration (0% and 10% salinity respectively) survived starvation longer than those emerging from higher salinities. In this study, survival of starvation was used as a proxy for energy reserves. Developing in saline rearing media had effect on how much energy reserve adult mosquitoes carry over from the larval stage. Adults emerging from L3 larvae reared in freshwater and 10% seawater survived for 72 hours, about three times longer than those reared in 20% and 30% seawater, which survived for only 24 hours. A similar trend was observed for L4 with larvae reared in freshwater and 10% seawater surviving for 96 hours, about four times longer than those reared in 20% seawater. This implies that those adults emerging from freshwater and lowest salinity possessed more energy reserves than those from higher salinities. The energy carried from the larval stage to adult stage is critical for survival since after emergence, adults depend on these energy reserves to search for sugar sources (Figure 6).

### 4.5. Morphometric Study of Adults Emerged from Larvae Exposure to Salinity

Rearing third and fourth instars in different concentrations of seawater appeared to have some effect on the wing length of resulting adults. The wing length of adults emerging from third instar (*P* = 0.031, *F*_34_ = 3.356) and fourth instar larvae (*P* = 0.017, *F*_17_ = 5.412), respectively, reared at the different concentrations showed significant differences (Table 1).

## 5. Conclusion

*An. coluzzii* demonstrated the clear preference for oviposition in freshwater though they are able to oviposit in water at all levels of salinity. However, egg hatchability, larval development, and energy reserves carried over from larval stage to adult were affected by higher salinities. This notwithstanding, *An. coluzzii* was able to breed successfully in saline water levels that fall within the range of brackish conditions. Such conditions exist in the coastal lagoons and estuaries that are abundant along the coast of Ghana as well as many other coastal countries on the West African corridor. Similarly, rising sea levels along the coast of Ghana (Keta area) and Nigeria (Lagos) can result in the creation of these brackish sites suitable for the proliferation of this species. This demonstrated ability to adapt to brackish water for breeding, is indicative of a clear possibility of *An. coluzzii* to extend its range to those areas. In addition, *An. coluzzii* populations may not be pushed out of their range because of salinization of coastal freshwater bodies resulting from climate change or rice fields resulting from evapotranspiration.

## Figures and Tables

**Table 1:** Morphometrics of adult *An. coluzzii *emerging from third and fourth instar larvae reared at different levels of seawater concentration.

Seawater concentration (%)	Wing length (cm)	
	L3	L4
0	2.96 ± 0.06^ab^	2.77 ± 0.12^a^
10	3.08 ± 0.05^b^	2.94 ± 0.07^a^
20	2.81 ± 0.08^a^	3.00 ± 0.04^a^
30	-	2.96 ± 0.04^ab^

Means in the same column with same small case alphabets as superscripts are not significantly different from each other at the 0.05 confidence level using Tukey's test.

## Data Availability

The data used to support the findings of this study are available from the corresponding author upon request.
